# Final year undergraduate nursing and midwifery students’ perspectives on simulation-based education: a cross-sectional study

**DOI:** 10.1186/s12912-022-01084-w

**Published:** 2022-11-06

**Authors:** Mairead Moloney, Louise Murphy, Liz Kingston, Kathleen Markey, Therese Hennessy, Pauline Meskell, Sandra Atkinson, Owen Doody

**Affiliations:** 1 Department of Nursing and Midwifery, University of Limerick, Limerick, Ireland; 2 Health Research Institute, University of Limerick, Limerick, Ireland

**Keywords:** Simulation-based education, Simulation-based learning experience, Nursing and midwifery education, Student-centred

## Abstract

**Background:**

Simulation-based education is a teaching and learning approach that can enhance learning experiences for students on healthcare programmes. Within undergraduate nursing and midwifery education, simulation can support students in developing graduate attributes necessary to become practice-ready professionals. This paper reports on the evaluation of a simulation-based education initiative, which was introduced to support final year undergraduate nursing and midwifery students in preparation for their upcoming clinical internship in practice.

**Methods:**

This study aimed to evaluate a simulation-based education initiative from the perspectives of final year undergraduate nursing and midwifery students (*N =* 95). An online survey, using the validated *Simulation Effectiveness Tool – Modified (SET-M),* was distributed to final year nursing and midwifery students at one university in Ireland. This study was conducted and reported in line with the Consensus-Based Checklist for Reporting of Survey Studies (CROSS).

**Results:**

The results of the study highlight final year nursing and midwifery students’ perceptions, experiences, and satisfaction with learning in a simulated environment. Students reported their simulation-based learning experiences as worthwhile, motivating, and as important opportunities to build on previous learning, increase confidence and gain experience in preparation for real-life practice. Students reported feeling more confident in their assessment skills, in providing care and interventions in responding to changes in a person’s health status. All students reported that the simulation-based learning experiences enabled them to think more critically about the clinical case scenarios and critically question their actions and decision-making processes. Pre-briefing and debriefing sessions were highlighted as important aspects of the simulation which helped to increase student confidence and cultivate meaningful learning.

**Conclusion:**

Simulation-based education is a valuable teaching and learning modality, particularly for final year students who are transitioning to real-life clinical practice. Student-centred simulation-based learning experiences can cultivate professional development and support learners in their transition from university student to healthcare professional.

**Supplementary Information:**

The online version contains supplementary material available at 10.1186/s12912-022-01084-w.

## Background

Simulation-based education has a long history as a teaching and learning modality in healthcare education. Within nursing and midwifery education, the goal of simulation is to prepare students for the transition into real-life clinical practice [1]. Simulation is effective in building critical thinking, clinical decision-making, and problem-solving skills, thereby facilitating the development of graduate attributes needed for practice-ready professionals [2, 3]. The clinical aspect of healthcare education is recognised as essential for students to engage and learn about healthcare provision [4] and clinical learning experiences are considered an essential aspect of quality education [5]. The transition from student to registered nurse/midwife is challenging and Kramer’s [6] seminal work referred to a “reality shock” as newly qualified nurses/midwives begin their career, and this evidence remains a reality [7, 8]. However, simulation-based education and clinical internships in healthcare settings provide valuable opportunities for adjustment and preparation for practice, enabling nursing/midwifery students to develop as clinicians [9–11]. Thus, integrating simulation-based education into pre-registration education supports students in gaining the necessary skills for autonomous, safe and competent practice, but most importantly students develop their own confidence for practice [1, 12].

This paper reports on the use of simulation-based education to support final year nursing and midwifery students transitioning from university-based learning to clinical internships in real-life healthcare practice. The simulation initiative encompassed a systematic, standardised approach to embedding simulation pedagogy within the students’ educational programme. A unique aspect of this initiative was the multi-stakeholder approach in the design and delivery of the learning experiences. This approach involved lecturers, clinical partners across four healthcare sites, educational technologist in simulation and student representatives across the undergraduate programmes. Simulation-based learning experiences were implemented across four undergraduate programmes, in year-four modules, over 7 weeks of clinical skills teaching aimed at actively engaging and preparing students for clinical internship. The initiative was underpinned by international Healthcare Simulation Standards of Best Practice [13]. This student centred, technology enhanced, structured pedagogical approach focused on active, collaborative, experiential and flexible learning using real-life co-created clinical scenarios, high fidelity mannequins and standardised participants (i.e., actors). Through the audio-visual recording of the simulation-based learning experiences, student learning was actively facilitated using debriefing strategies such as reflection, self-assessment, and peer performance review. Unique considerations which greatly authenticated this initiative were the co-creation of realistic clinical scenarios between students, lecturers and clinical partners, in tandem with critical application of technology to enhance teaching and learning practices to promote greater student engagement.

It is acknowledged that students in this study had past exposure to simulation pedagogy throughout their four-year undergraduate nursing or midwifery programmes. However, this exposure was mostly an under-recognised and informal educational process (e.g., simulation role plays, case studies), and usually implemented at the discretion of individual lecturers/teachers without formal support. As an enabler of success in this study, the importance of the support offered by the multistakeholder approach, engagement with the dedicated simulation team and the adoption of simulation standards of best practice in relation to the design and implementation of simulation-based education cannot be overstated. It is also noteworthy that the implementation of this initiative was impacted by the Covid-19 pandemic, which resulted in scheduled clinical placement opportunities for students being limited or cancelled. Thus, this initiative was timely, as it offered students an opportunity to prepare for practice in a safe environment and the findings highlight the value of implementing formal, structured simulation-based learning experiences particularly for final year students who are preparing for professional clinical practice in a continuously evolving, complex healthcare environment.

The objective of this study was to evaluate students’ perspectives of the simulation-based learning experiences and this paper reports on a cross-sectional survey conducted as phase one of a mixed methods study. The Modified Simulation Effectiveness Tool (SET-M) survey instrument (19 items) was used as it is a validated tool for evaluating students’ perceptions on the effectiveness of simulation in relation to meeting their learning needs. The SET-M has very good internal reliability, with a Cronbach’s alpha for the overall SET-M survey instrument reported as α = .936 [14].

## Methods

### Setting

This study was conducted in a university setting in the West of Ireland. A quantitative cross sectional survey design involving an online questionnaire was used to survey final year undergraduate nursing and midwifery students who had participated in simulation-based learning experiences.

### Ethical considerations

The study was performed in accordance with the Declaration of Helsinki and ethical approval was granted by the *Education and Health Sciences Research Ethics Committee* (EHSREC No: 2020_06_17_EHS) at the originating university. All involved in the study were bound by national and international codes of good practice in research, and by professional standards. The Head of Department was approached with details of the study and permission was granted to access and invite participants. An independent staff member sent a recruitment email, study information sheet and survey link to the participants via the university email system. It was clearly communicated that participation was voluntary, and that any data gathered would be anonymous and treated confidentially in line with General Data Protection Regulations (GDPR). Informed consent was obtained from all participants prior to participating. It is recognised that power dynamics exist in all kinds of relationships, particularly so in the teacher-student relationship [15], so in an attempt to address power dynamics and to protect anonymity, the student details (email address) or any identifying information were not linked to the survey responses. Participation was voluntary and data gathered was treated confidentially in line with General Data Protection Regulations (GDPR).

### Participants

A purposeful non-random convenience sample was chosen, and the sample population included all final year (i.e. year four) undergraduate students (*N =* 95) across four programmes - BSc Nursing (Intellectual Disability), BSc Nursing (Mental Health), BSc Nursing (General) and BSc Midwifery from one university in Ireland. In total 67 students out of a possible 95 responded to the survey (response rate 70.5%).

### Data collection

The modified Simulation Effectiveness Tool (SET-M) survey instrument (19 items) was utilised for data collection, along with an addition subscale developed by the researchers to measure students learning experience (7 items), demographics (1 item) and open-ended questions (1 item). The Simulation Effectiveness Tool (SET) was originally developed in 2005 and later modified (SET-M) to align with international healthcare simulation standards of best practice, such as those published by the International Nursing Association for Clinical Simulation and Learning (INACSL), Quality and Safety Education for Nurses (QSEN), and the American Association of Colleges of Nursing [14]. Questions are designed to assess the effectiveness of simulation experiences in healthcare education. The SET-M is considered a reliable and valid approach to measuring effectiveness of learning through simulation and is psychometric tested [14]. There is a very good level of internal reliability and consistency reported in the original study for the overall survey (Cronbach’s alpha α = .936) and individual subscales (Pre-briefing α = .833, Learning α = .852, Confidence α = 913, Debriefing α = .908) [14].

The rationale for adding a sub-scale developed by the researchers to the SET-M (subscale to measure students learning experience/7 items, demographics/1 item and open-ended questions/1 items) was to identify any patterns or potential relationships within or across nursing disciplines and midwifery and to gather students’ perspectives. These items were chosen as they represented general demographic questions used in similar surveys and were also used in previous studies by the research team. The subscale was agreed by the research team and student representatives leading to further face and content validity. Also, some logistical questions were used to gather information about the timing and duration of the sessions (4 items) to allow consideration for future sessions and support quality improvement. An additional response option of N/A (i.e., not applicable) was added to some questions, the rationale being that the question may not have been applicable to the simulation scenario undertaken in the specific discipline (e.g., mental health, general, intellectual disability nursing or midwifery).

For this study, the survey involved an online version of the SET-M tool, generated using Qualtrics ^xm^ software [16]. An anonymous survey hyperlink was included in the recruitment email, which was administered in November 2020 and remained live for 30 days. This gave potential participants time to carefully consider their participation. The survey consisted of open and closed questions and took approximately 10 minutes to complete, this was clearly communicated in the recruitment email as the authors were cognisance that completion time and participation rates correlate [17]. The IP address of each participant’s computer was used to identify potential duplicate entries from the same user. There was a time period of 24 hours for which no two entries from the same IP address were allowed.

### Data analysis

The survey data were transferred to IBM SPSS v. 26, screening for missing data and descriptive statistics were performed using frequencies and percentages. Cronbach’s alpha coefficient was calculated for the overall survey and for the individual subscales of the survey. In some previous studies the individual subscales of the SET-M were sub-divided, and these values were reported e.g., scenario subscale is subdivided into learning and confidence. Alpha ranges from 0 to 1, with higher values indicating that the survey is more reliable. The acceptable range for Cronbach’s Alpha in this study was deemed to be 0.7 and above, with any values lower than 0.7 considered poor internal consistency, and values greater than 0.8 considered to indicate good internal consistency [18]. In relation to missing data, the majority of questions were answered on the survey and any missing data was omitted from analysis. Qualitative data arising from the open-ended survey questions were analysed using content analysis guided by Colorafi and Evans [19] steps of (1) create a coding framework, (2) add codes and memos, (3) apply the first level of coding, (4) categorise codes and applying the second level of coding, (5) revise and redefine codes, (6) add memos, (7) visualise data and (8) represent the data. This approach allowed for the systematic identification and organisation of patterns of meaning (codes, categories and themes) across the data set. Two members of the team (MM, OD) identified initial codes and categories and agreement was reached by meeting to discuss, a third member of the team (LM) was available if any discrepancies could not be resolved. The quantitative results are presented initially followed by a summary of the qualitative findings. The study was conducted and reported in line with the Consensus-Based Checklist for Reporting of Survey Studies – CROSS [20] (Supplementary file 1).

## Results

The results section presents the internal consistency reliability of the SET-M instrument which is outlined in Table 1. This is followed by the reported demographics, in order to provide context to the study. In addition, a narrative of descriptive statistics including frequencies and percentages for the three sections of the SET-M individually with an overall tabular representation in Table 2, with the additional subscale presented in Table 3. Each individual subscale of the SET-M and the instrument overall was assessed for internal consistency reliability. The three individual subscales (pre-briefing, scenario, debriefing,) of the SET-M and the additional subscale added in this study are outlined in Table 1 in addition to the number of items, related questions and Cronbach alpha. The internal reliability for the three individual subscales was good ranging from .739–.997. The SET-M’s overall internal consistency reliability was good (α = .824) which was similar to the original study (α = .936) and the overall SET-M instrument (Q1-Q19) combined with the additional subscale of application to practice (Q20-Q26) had an overall good internal consistency reliability (α = .907). The Cronbach alphas for the subscales in this study (except the additional subscale added in this study) are compared to scores from the original study [14] and a recent study by Bergamasco and Cruz [21] in Table 1. Cronbach alphas for two domains (pre-briefing, debriefing) had a similar internal consistency in comparison to the developers’ original study [14] and were slightly higher than Bergamasco and Cruz’s study [21]. However, within the scenario subscale researchers report a breakdown of this subscale into its learning and confidence components and within this study learning and confidence were reported as (α = .557, .658) respectively. While these Cronbach’s alphas were poor, the overall scenario subscale was acceptable (α = .739). The additional subscale application to practice consisted of seven items (Q20-Q26) and internal consistency reliability was good (α = .997).

**Table 1:** Internal Consistency Reliability for original SET-M compared with other studies

SET-M Sub Scales	Number of Items	Related Questions	Current Study Cronbach’s Alpha α	Benchmark data from Leighton et al. (2015) original study Cronbach’s Alpha α	Data from Bergamasco and Cruz (2021) Study Cronbach’s Alpha α
**Pre-briefing**	2	Q1, Q2	.827	.833	.792
**Scenario**	12	Q3-Q14	.739	Not reported	Not reported
Learning	6	Q3-Q8	.557^a^	.852	.823
Confidence	6	Q9-Q14	.658^a^	.913	.874
**Debriefing**	5	Q15-Q19	.888	.908	.758
**Application to Practice**	7	Q20-Q26	.997	N/A	N/A
**Total Original SET-M Survey**	19	Q1-Q26	.824	.936	Not reported in paper

^a^poor reliability

**Table 2:** Students (*n =* 67) responses to SET-M survey questions

	Strongly Agree N (%)	Somewhat Agree N (%)	Do Not Agree N (%)	Not Applicable N (%)	Missing Data N (%)
**Prebriefing**					
Q1 Prebriefing increased my confidence	57 (85.1%)	9 (13.4%)	1 (1.5%)	0 (0%)	0 (0%)
Q2 Prebriefing was beneficial to my learning	59 (88.1%)	6 (9%)	2 (3%)	0 (0%)	0 (0%)
**Scenario**					
Q3 I am better prepared to respond to changes in the patient/woman’s condition.	47 (70.1%)	20 (29.9%)	0 (0%)	0 (0%)	0 (0%)
Q4 I developed a better understanding of the pathophysiology ~ (Leave blank if not relevant).	26 (38.8%)	19 (28.4%)	2 (3%)	4 (29.9%)	0 (0%)
Q5 I am more confident of my assessment skills.	45 (67.2%)	22 (32.8%)	0 (0%)	0 (0%)	0 (0%)
Q6 I felt more confident in my ability to make clinical decisions.	40 (59.7%)	26 (38.8%)	1 (1.5%)	0 (0%)	0 (0%)
Q7 I developed a better understanding of medications ~ (Leave blank if no medications in scenario).	15 (22.4%)	20 (29.9%)	3 (4.5%)	29 (43.3%)	0 (0%)
Q8 I had the opportunity to practice my clinical decision making skills.	48 (71.6%)	16 (23.9%)	3 (4.5%)	0 (0%)	0 (0%)
Q9 I am more confident in my ability to prioritise care and interventions.	40 (59.7%)	25 (37.7%)	2 (3%)	0 (0%)	0 (0%)
Q10 I am more confident in communicating with the patient/woman.	57 (85.1%)	10 (14.9%)	0 (0%)	0 (0%)	0 (0%)
Q11 I am more confident in my ability to teach the patient/woman about their illness and Interventions ~ (Leave blank if not relevant).	27 (40.3%)	19 (28.4%)	2 (3%)	19 (28.4%)	0 (0%)
Q12 I am more confident in my ability to report information to the health care team.	44 (65.7%)	22 (32.8%)	1 (1.5%)	0 (0%)	0 (0%)
Q13 I feel more confident in providing interventions that foster patient safety.	45 (67.2%)	21 (31.3%)	1 (1.5%)	0 (0%)	0 (0%)
Q14 I am more confident in using evidence-based practice to provide nursing/midwifery care.	50 (74.6%)	17 (25.4%)	0 (0%)	0 (0%)	0 (0%)
**Debriefing**					
Q15 Debriefing contributed to my learning.	58 (86.6%)	9 (13.4%)	0 (0%)	0 (0%)	0 (0%)
Q16 Debriefing allowed me to verbalise my feelings before focusing on the scenario.	52 (77.6%)	14 (20.9%)	1 (1.5%)	0 (0%)	0 (0%)
Q17 Debriefing was valuable in helping me improve my clinical judgement.	57 (85.1%)	10 (14.9%)	0 (0%)	0 (0%)	0 (0%)
Q18 Debriefing provided opportunities to self-reflect on my performance during simulation.	56 (83.6%)	10 (14.9)	1 (1.5%)	0 (0%)	0 (0%)
Q19 Debriefing was a constructive evaluation of simulation.	56 (83.6%)	11 (16.4%)	0 (0%)	0 (0%)	0 (0%)

~ Not Applicable Response Option is only applicable to Q4, Q7 and Q11

**Table 3:** Students (*n =* 67) responses to additional subscale on application to practice

	Strongly Agree N (%)	Somewhat Agree N (%)	Do Not Agree N (%)	Missing N (%)
Q20 The simulation will enable me to think more critically about the clinical case scenario.*64	58 (86.6%)	6 (9%)	0 (0%)	3 (4.5%)
Q21 The simulation will enable me to become more aware of the evidence behind my practice. *64	56 (83.6%)	8 (11.9%)	0 (0%)	3 (4.5%)
Q22 The simulation will enable me to critically question my decision-making processes. *64	57 (85.1%)	7 (10.4%)	0 (0%)	3 (4.5%)
Q23 The simulation will enable me to become more reflective of my own practice. *64	57 (85.1%)	7 (10.4%)	0 (0%)	3 (4.5%)
Q24 My learning from the simulation will have a positive impact on the care I deliver. *64	59 (88.1%)	4 (6%)	1 (1.5%)	3 (4.5%)
Q25 My learning from the simulation can be applied to my future career as a nurse/midwife. *64	58 (86.6%)	6 (9%)	0 (0%)	3 (4.5%)
Q26 The simulation will enable me to be more confident in communicating with my colleagues/healthcare team members. *64	58 (86.6%)	5 (7.5%)	1 (1.5%)	3 (4.5%)

* Number — * = missing data, number = actual responses

### Demographics

Of the target survey population (*N =* 95), 67 final year undergraduate nursing and midwifery students completed the survey (70.5% response rate), of which 41.8% (*n =* 28) were General, 25.4% (*n =* 17) Mental Health, 20.9% (*n =* 14) Intellectual Disability nursing students and 11.11% (*n =* 8, 80% response of midwifery students) Midwifery students. All students (*n =* 67) had one-hour simulation-based learning experience sessions. Of these sessions, 85.1% of students (*n =* 57) strongly agreed and somewhat agreed that the one-hour timing of the simulations was just about right, with 10 students (14.9%) who did not respond to the question. In addition, 98.5% of students (*n =* 66) across the programmes strongly agreed and somewhat agreed that they would like to see more simulation-based learning experiences in their programme of study, with just one student (1.5%) who did not want more simulations.

### Pre-briefing

The majority of students reported that the pre-briefing session (Q1, Q2) prior to the simulation-based learning experience had increased their confidence and was beneficial to their learning (see Table 2). The majority of students agreed that pre-briefing increased their confidence and was beneficial to their learning (97.1%, *n =* 59).

### Scenario

The scenario subscale elicited students’ perceptions on overall learning and confidence (Q3-Q14). As illustrated in Table 2, all students reported that they were better prepared to respond to changes in the patients/woman’s condition as a result of the simulation, while the majority (67.2%, *n =* 45) reported they developed an improved understanding of pathophysiology. All students reported that they felt more confident in their nursing/midwifery assessment skills, while most students (98.5%, *n =* 67) reported that they felt more confident in their ability to make clinical decisions.

Regarding medications management, this question was only relevant to 56% students (*n =* 38) as not all students had medication management in their simulation scenarios. Of the relevant students, most (*n =* 35) reported that they developed a better understanding of medications after participating in the simulation-based learning experience. Most students (95.5%, *n =* 64) indicated that they had the opportunity to practice their clinical decision-making skills during the simulation-based learning experience, while 97.4% of students (*n =* 64) reported that they were more confident in their abilities to prioritise care and interventions. All students reported that they were more confident in communicating with the patient/woman.

Regarding patient education, most students (68.7%, *n =* 46) agreed that they had more confidence in their ability to teach the patient/woman about their illness/condition and interventions, with only two students that did not agree (3%) and 19 students (28.4%) that felt this question was non-applicable. The majority of students (98.5%, *n =* 66) reported that they were more confident in their ability to report information to the health care team and that they were more confident in providing interventions that foster patient/woman safety (98.5%, i66). In addition, all students stated that they were more confident in using evidence-based practice to provide nursing/midwifery care.

### Debriefing

All students reported that debriefing was a valuable component of the simulation-based learning experience which contributed to their learning and that debriefing was valuable in helping them improve their clinical judgement. The majority of students (98.5%, *n =* 66) agreed that debriefing allowed them to verbalise their feelings before focusing on a scenario and all students agreed that debriefing provided opportunities to self-reflect on their own performance during simulation.

### Application to future practice

Table 3 reports the results of seven additional questions the authors added to the survey to capture the application of the simulation-based learning experience to future practice. The majority of students (95.6%, *n =* 64) reported that the simulation-based learning experience will enable them to think more critically about the clinical case scenario. Similarly, all students reported that the simulation-based learning experience will enable them to become more aware of the evidence behind their practice, with only three students that did not respond to the question. Most students (95.5%, *n =* 64) stated that simulation-based learning experience will enable them to critically question their decision-making processes.

Most students (*n =* 64) felt that the simulation-based learning experience will enable them to become more reflective of their own practice, with only three students that did not respond to the question. The majority of students (*n =* 63) reported that their learning from the simulation-based learning experience will have a positive impact on the care that they deliver (94.1%, *n =* 63). Most students (97.6%, *n =* 64) stated that their learning from the simulation-based learning experience can be applied to their future career as a nurse/midwife. The majority of students agreed (94.1%, *n =* 63) that the simulation-based learning experience will enable them to be more confident in communicating with their colleagues/healthcare team members, with only one student that did not agree.

### Qualitative responses

Participants were afforded the opportunity via open comments to identify or express anything else they would like to add about their simulation-based learning experience. There were 18 responses, representing 26.86% of the 67 survey respondents. Through content analysis guided by Colorafi and Evans [19] categories were generated. The first category was ‘effective learning experience*’* and within this, students expressed positive views on simulation as a learning modality. Students enjoyed the session, found it fun, relevant, beneficial, and worthwhile and particularly enjoyed the interaction between peers and lecturers as described by one participant ‘*the stimulation lab was fun and interactive and really got me thinking and motivated in my studies, I really enjoyed the interaction between lectures and students’*. The second category identified was *‘*opportunity to prepare for real life clinical practice*’*, students found the simulations realistic, motivating, and viewed it as an important opportunity to build on previous learning, increase confidence and gain experience in preparation for clinical internship, for example one participant explained ‘*it was very helpful and allowed me time to reflect on what I would do in an emergency situation, it was reassuring for returning back to placement’.* Students also spoke of the opportunity for reflection and learning via constructive feedback from peers and lecturers. The final category generated highlighted suggestions for *‘*enhancing the student experience’, some reported uncertainty about their role expectations on the day and expressed feeling unprepared for the simulation. In relation to performing in front of peers during the simulation, some expressed feelings of embarrassment, awkwardness and intimidation, for example one participant stated ‘*I felt a bit awkward and unnatural, I am unsure how to overcome this’*. It was suggested that more time and attention to the pre-briefing stage of simulation may help address these issues for students in future.

## Discussion

Within this study, and similar to other published work, it was evident that students felt simulation was an educational tool capable of simulating real clinical situations in a safe environment, which enabled them to develop cognitive, psychomotor and affective skills [22–27]. Furthermore, supporting students to become critical thinkers, independent decision-makers, lifelong learners, effective team members and competent users of new information technologies [28]. Within students’ learning experiences, the sense of safety is important as it allows students to practice and correct their mistakes without risks to patients and with minimal risk to themselves [29]. Thus, simulation allows for development and exchange of ideas, teamwork and team leadership, creative thinking and problem-solving situations that focus on engagement and motivation of the learner [23, 27, 30–33]. Given the high agreement level achieved in the SET-M in this study it is anticipated that simulation prepares students for their future practice roles.

Most nursing and midwifery education programmes require students to complete a clinical internship in healthcare settings to gain practical experience and integrate knowledge and clinical skills. This experience is considered a transition from academic education to a professional career [5] and during this transition, students often lack confidence and this affects their ability to work in the complex healthcare field [34]. Transitioning to nursing or midwifery practice is recognised as challenging [34–37]. In contemporary times, safety concerns such as the covid-19 pandemic and increasingly complex dynamic healthcare environments, have resulted in restricted or reduced opportunities for nursing and midwifery student experiences [38]. These limitations impact nursing and midwifery students’ abilities to engage in learning opportunities that support the development and advancement of the skills needed for their future practice such as delegation, prioritisation, care management, independent decision-making and interprofessional collaboration [39]. Thus, preparing the next generation of graduating nurses/midwives requires innovative and transformative educational experiences [36, 39], to overcome the transitional challenges that lie ahead. Simulation-based education is one solution which places nursing and midwifery students in a position of leadership to independently assess, make decisions, and manage all aspects of patient care, nurturing strong assessment skills, clinical judgment skills, delegation, prioritisation, and time management [34, 35, 37, 39, 40]. This was evident in this study, by the high level of agreement for teamwork and decision making (98.5%) and management skills (95.5%). Exposure to simulation-based education has an important role in grounding students’ experiences and preparing them for the transition to their future nursing and midwifery roles. Thus, addressing the anticipated ‘reality shock’ which is known to occur during transition.

There is ample evidence in the literature that simulation-based education is a creative and engaging strategy that is increasingly incorporated into nursing and midwifery curricula internationally [1, 41, 42]. Simulation is a dynamic opportunity for integrating interactive learning strategies, problem-based learning and case-based learning [43], which enables students to gain the confidence, knowledge and skills required for their current and future practice [44] in a non-threatening, safe environment [45]. Thus, creating an environment that supports students with a transformative learning experience, is particularly important for those transitioning to real-life clinical practice. Fundamental to successful integration of simulation-based education are careful planning, preparation and adherence to internationally recognised best practices standards regarding design, implementation and facilitation. For successful learning to occur, simulations should be planned in a way that takes cognisance of the complexity of practice scenarios and demands in practice, in a manner that supports students in acquiring skills gradually [46]. Within the planning of scenarios, a co-creation philosophy with stakeholders is important, one that takes account of the facilitators, students and clinical partners’ perspectives in attempt to improve the clinical integrity of the scenarios, which subsequently can be adjusted to the learner’s level of development [47, 48]. This is essential given that the quality of care expected by patient safety standards and citizens themselves require a high level of professional qualifications, skills and safety to promote the welfare of society [49]. Similarly, the education, preparation and skills of those facilitating simulation and the dynamics of the relationship between facilitator and students needs to be recognised [50]. In this study, cognisant of the importance of planning, stakeholder engagement was an important aspect in the design and development of the scenarios used within the simulation-based learning experiences. This engagement process created and ensured fidelity and authenticity as close as possible to the real world experience.

Evident within this study was the importance of the pre-briefing and debriefing in the overall student learning process. Pre-briefing and debriefing are considered essential features of any formal, structured simulation-based learning experience [41, 51] and the recently published *Healthcare Simulation Standards of Best Practice™* [13] highlight their importance in the evidence-based guidelines for best practice standards in simulation. Pre-briefing relates to the preparation and briefing that students’ receive in advance of the simulation-based learning experience [51]. Successful pre-briefing enables students to approach their learning experiences with clear instruction regarding their role and performance expectations, and in addition it generates an awareness for students around ground rules. Debriefing, in this educational context, refers to a planned facilitator-guided process usually carried out immediately after the event, and can include a range of activities such as providing feedback, discussion and guided reflection [52]. Effective debriefing allows students the opportunity to explore emotions and experiences in a professional environment to better understand concepts, ideas, or behavioural insights [53]. Pre-briefing and debriefing are particularly important in simulation-based education, as similar to any experiential-based learning strategy, learning is dependent on bridging the actual experience and the cognitive insights associated with that, in order to create a meaningful learning experience and to identify knowledge gaps and potential areas of future development for the learner [1, 52, 53]. Such a formalised teaching approach ensures formality, structure and support around simulation design and implementation, which in turn helps to champion its integration within nursing and midwifery curricula. Internationally, equal weighing is given to both theory and practice within nursing and midwifery educational curricula, therefore emphasising the necessity to provide authentic experiences within the delivery of the theoretical elements of curricula is important and simulation-based education can offer opportunities here. This approach enables the theory to be grounded and translated to practice and can be provided across all nursing and midwifery skills e.g., technical and non-technical skills.

To promote a comprehensive standard of practice in simulation-based education, it is important that adequate time and attention is afforded to simulation design and implementation. As with all educational strategies, these processes need to be grounded in theoretical frameworks and/or evidence-based concepts and ideally facilitated by those experienced in simulation-based education [54, 55]. While students in this study indicated they were in favour of participating in future simulation-based learning experiences, it must be recognised that simulation requires a significant investment of time, money and resources [56]. To ensure the success of simulation-based education across nursing and midwifery programmes, a sustainable, cohesive strategic approach regarding the financial planning, provision of resources, and the education of simulation facilitators is needed.

### Limitations

The limitations of this study are primarily related to the sample size and the fact that the study was conducted in a single institution. There was a good response rate of 70.5% for the survey, however, there were some limitations to data collection. The findings from the survey open-ended questions represent 26.86% (*n =* 18) of the 67 responses and this should be considered when interpreting results. It is important to emphasise that the study focussed on the students’ ‘perceptions’ of the impact of simulation and therefore it is difficult to claim any impact on their future practice. In addition, no pre/post-test design or control group to measure the impact were included in this study. However, validity in this study was addressed through the use of an existing tool with construct, content and criterion validity and internal reliability for the study was good. Further research with many participants and multiple settings/locations is needed to determine the effectiveness of simulation.

## Conclusion

Student-centred simulation-based learning experiences cultivate meaningful learning opportunities for undergraduate nursing and midwifery students, particularly so in contemporary times when the availability and learning conduciveness of real-life clinical learning environments is limited. Supporting final year students, transitioning from university student to healthcare professional, can be enhanced with simulation-based learning experiences which foster the development of the graduate attributes needed by practice-ready professionals. The evidence and possibilities for using simulation-based education within undergraduate programmes to minimise the theory-practice gap and prepare students for dynamic fast-paced healthcare environments is irrefutable. However, there is a need for a more sustainable, cohesive and strategic approach regarding the implementation of quality simulation-based initiatives across nursing and midwifery programmes. This paper offers an example of a systematic process for embedding simulation-based education in undergraduate programmes by advocating a multistakeholder approach which includes lecturer, student and clinical partner collaboration, in tandem with a dedicated simulation team committed to the continuous enhancement of student success. In the transformative world of healthcare education, simulation is an essential educational approach which can empower students to become professionals capable of providing patient-centred, safe care of the highest standards.

## Supplementary Information

**Additional file 1.**

## Data Availability

All data generated or analysed during this study are included in this published article and its supplementary information files.
